# Case Report: Testicular Torsion in Unilateral Supernumerary Testis

**DOI:** 10.3389/fped.2022.823374

**Published:** 2022-04-11

**Authors:** Lu Xiaofei, Zhou Benzhang

**Affiliations:** Department of Urology, Xiangyang No.1 People's Hospital, Hubei University of Medicine, Xiangyang, China

**Keywords:** polyorchidism, testicular torsion, supernumerary testis, case report, pediatrics–children

## Abstract

Supernumerary testis (SNT), or polyorchidism, is a rare congenital anomaly of the genitourinary tract, described as the presence of more than two testicles. Testicular torsion (TT) in unilateral SNT is extremely rare. We report a case of unilateral SNT in a 16-year-old boy accompanied by TT, which was diagnosed preoperatively based on the outcomes of physical examination and ultrasound and confirmed intraoperatively. We opted for orchiectomy of this SNT because of the long-time hypoperfusion. And as for the normal testis, orchidopexy was performed. His clinical presentation subsided after surgery, and the patient was discharged 3 days later.

## Introduction

Supernumerary testis or polyorchidism is a rare congenital anomaly of the genitourinary tract, described as the presence of more than two testicles. The majority of the patients do not have any clinical symptoms or are detected by imaging or during surgical explorations accidentally (1). Testicular torsion in unilateral supernumerary testis is extremely rare; around 30 cases have been reported in the literature up to the present (2). We report a case of unilateral supernumerary testis in a 16-year-old boy accompanied by testicular torsion, which was diagnosed preoperatively based on the outcomes of physical examination and ultrasound and confirmed intraoperatively.

## Case Report

A 16-year-old boy with a history of polyorchidism presented to our hospital with a 2-day history of left testicular and inguinal pain. He denied a history of scrotal trauma, and the patient did not feel any discomfort previously with the history of polyorchidism. On physical examination, the left side of the scrotum was markedly red and swollen, and two masses were identified in the left hemi-scrotum. The higher mass was painful, hard on palpation, and elevated compared to the right testicle. The lower mass was palpable in the left hemi-scrotum. The left cremasteric reflex was significantly diminished. After admission, his blood work was completed: leukocytes (7,620/μl), hemoglobin (16 g/dl), platelets (303,000/μl), LDH (173 co/L), HCG (1.2 mIU/ml), and AFP (2.15 ng/ml).

**Figure 1:**
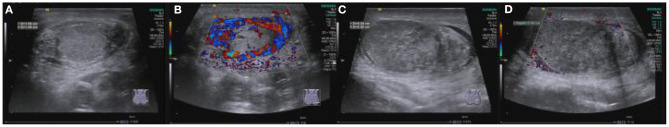
Color Doppler image: Two testicles were lying in the left hemi-scrotum. The lower testicle **(A)** is smaller than the higher testicle **(C)** and has normal blood flow **(B)**. There is no visible vascular flow in the higher testicle **(D)**.

An ultrasonography scan of the scrotum revealed two hypoechoic masses, both with testicular-like textures. The higher mass, measuring 3.1 × 2.0 cm, did not have visible vascular flow inside on color Doppler examination (Figures 1C,D); the lower mass, measuring 1.9 × 1.5 cm, had visible vascular flow (Figures 1A,B). Based on the outcomes of physical examination and ultrasound, the patient was diagnosed with polyorchidism and testicular torsion in the left hemi-scrotum. Therefore, emergency scrotal exploration was performed under spinal anesthesia. The patient's parents were informed of the possibility of orchiectomy in surgery.

**Figure 2:**
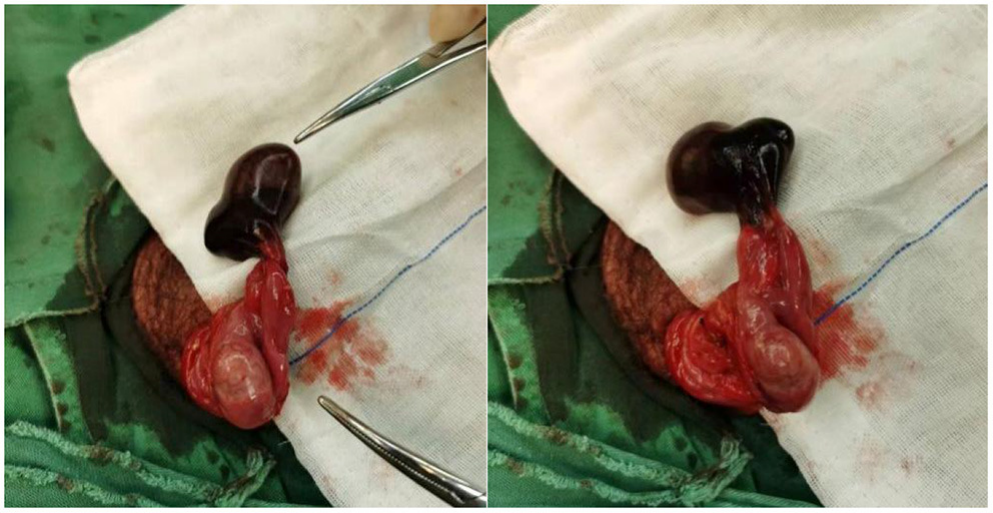
Emergency scrotal exploration was performed; the upper left testicle was black and twisted 360° clockwise. The lower left testicle was normal in its color.

During operation, we found that two testicles were lying within the single tunica vaginalis. The upper left testicle was black and twisted 360° clockwise. The lower left testicle was normal in its color. On gross examination, the two testicles share the same vas and two separate epididymites (Figure 2). Detorsion was performed on the black testis; after 15 min of observation, the testicle color was not changed obviously. No blood was found after incision of the tunica albuginea. Thus, we decided to perform a left orchiectomy of the larger testis. And as for the normal ipsilateral testis and the contralateral testis, orchidopexy was performed. The operation lasted 45 min; the patient was discharged on the third postoperative day. Routine surveillance was advised.

## Discussion

Polyorchidism is a rare congenital anomaly of the genitourinary tract. Around 200 cases have been reported up to the present after the first cases reported by Blasius in 1670, and triorchidism is the most common type (1). The supernumerary testis commonly appears on the left side of the scrotum, which was occurs in our patient (1, 2). Most patients do not have any clinical symptoms or are detected by imaging or during surgical explorations accidentally. And more than 40% of patients with polyorchidism have a history of undescended testicles discovered in their early childhood (1–3).

The precise etiology of polyorchidism is unknown. A literature review showed that the testes occur early in the sixth week of ovulation age and originate from the primitive genital ridge. However, the vas deferens and epididymis develop from the mesonephric duct 2 weeks later. The most popular theory is a transverse division of the genital ridge that can clarify all of the forms of supernumerary testis (1–3). Based on embryological development, Leung et al. divided polyorchidism into four types: Type 1, supernumerary testis only, no epididymis, no vas deference; Type 2, supernumerary testis, shares one epididymis and vas deference with ipsilateral testis; Type 3, supernumerary testis with own epididymis, shares one vas deference; Type 4, every supernumerary testis has its own epididymis and vas deference.

Type 2 is the most common of the four types (2). According to the classification criteria of Leung, our patient belongs to Type 3 of polyorchidism.

So far, there are no guidelines for managing polyorchidism because of the rare incidence that has been reported until now. Most experts recommend a conservative approach to patients with supernumerary testes that had descended to the scrotum and been confirmed by imaging such as ultrasound or magnetic resonance imaging. Regular ultrasound follow-up is needed (4). If the undescended supernumerary testis was detected during surgical explorations accidentally, orchiectomy could be performed for Type 1 testes because of its uselessness in fertility (4, 5). But for other types of polyorchidism, most experts believe that orchidopexy should be performed for the undescended supernumerary testis for its fertility potential. But others argue that the incidence of testicular malignancy in the undescended supernumerary testis was much higher than in the normal population (7 vs. 0.004%), and the majority of supernumerary testes have spermatogenesis obstacle. So orchiectomy should be performed for all undescended supernumerary testes (6–8).

Testicular torsion in unilateral supernumerary testis that has descended to the scrotum is extremely rare: around 30 (about 12%) cases have been reported in the literature until the present (2). Testicular torsion is one of the urological emergencies with peak incidence at puberty and requires emergency repair (9). Currently, the diagnosis of testicular torsion is mainly based on history, physical examination, and imaging sign. Testicular torsion accounts for only 25% of cases suspected for testicular torsion with acute scrotal pain preoperatively (10). On physical examination, the typical symptom is a painful, enlarged, hardened, and high-riding testis. Color Doppler ultrasound is the best tool to diagnose testicular torsion (11). But Abbas et al. argued that there is no single clinical or imaging sign that can prove or rule out testicular torsion (12). Emergent exploration is recommended when testicular torsion is suspected (10, 12, 13).

In our case, intravaginal torsion occurred in the supernumerary testis. Beiko et al. (14) believe that the supernumerary testis is more prone to develop torsion because of the absence of gubernaculum, which was a normal attachment between the epididymis and scrotal wall. In our case, we found there is no attachment between the supernumerary testis epididymis and the scrotal wall. In addition, the longer pedicle of the supernumerary testis increases the activity of the testis, which increases the incidence of intravaginal testicular torsion.

In our case, the supernumerary testis has separate epididymis and shares the same vas with the normal testis in the hemi-scrotum. The fertility potential was considered, but we opted for orchiectomy for this supernumerary testis because of the nonvital testicle. Feher et al. (15) found that the duration of symptoms and degree of twisting were the two factors affecting the viability of the twisted testis through a study of the published literature for nearly 5 years. If the symptoms last beyond 6 h, they believe the viability of the twisted testis will decline significantly. So for patients with suspected testicular torsion, recognition and emergency scrotal exploration are needed as soon as possible.

## Conclusion

Supernumerary testis is a rare congenital anomaly of the genitourinary tract, but it is a high-risk factor of testicular torsion. Active surveillance is necessary; if testicular torsion occurs, early and accurate diagnosis can reduce the chance of orchiectomy for nonvital testicles.

## Data Availability Statement

The original contributions presented in the study are included in the article/Supplementary Material, further inquiries can be directed to the corresponding author.

## Ethics Statement

Written informed consent was obtained from the individual(s) and minor(s)' legal guardian/next of kin, for the publication of any potentially identifiable images or data included in this article.

## Author Contributions

LX: writing—original draft preparation. ZB and LX: writing—review and editing. All authors have read and agreed to the published version of the manuscript.

## Conflict of Interest

The authors declare that the research was conducted in the absence of any commercial or financial relationships that could be construed as a potential conflict of interest.

## Publisher's Note

All claims expressed in this article are solely those of the authors and do not necessarily represent those of their affiliated organizations, or those of the publisher, the editors and the reviewers. Any product that may be evaluated in this article, or claim that may be made by its manufacturer, is not guaranteed or endorsed by the publisher.
